# Laparoscopic cholecystectomy for acute calculous cholecystitis: a retrospective study assessing risk factors for conversion and complications

**DOI:** 10.1186/s13017-016-0111-4

**Published:** 2016-11-16

**Authors:** Petra Maria Terho, Ari Kalevi Leppäniemi, Panu Juhani Mentula

**Affiliations:** 1 Institute of Clinical Medicine, Faculty of Medicine, University of Helsinki, Haartmaninkatu 8, 00014 Helsinki, Finland; 2 Department of Abdominal Surgery, Helsinki University Central Hospital, P.O.Box 340, 00029 HUS Helsinki, Finland

**Keywords:** Acute cholecystitis, Laparoscopic cholecystectomy, Open cholecystectomy, Conversion

## Abstract

**Background:**

The purpose of the study was to identify risk factors for conversion of laparoscopic cholecystectomy and risk factors for postoperative complications in acute calculous cholecystitis. The most common complications arising from cholecystectomy were also to be identified.

**Methods:**

A total of 499 consecutive patients, who had undergone emergent cholecystectomy with diagnosis of cholecystitis in Meilahti Hospital in 2013–2014, were identified from the hospital database. Of the identified patients, 400 had acute calculous cholecystitis of which 27 patients with surgery initiated as open cholecystectomy were excluded, resulting in 373 patients for the final analysis. The Clavien-Dindo classification of surgical complications was used.

**Results:**

Laparoscopic cholecystectomy was initiated in 373 patients of which 84 (22.5%) were converted to open surgery. Multivariate logistic regression identified C-reactive protein (CRP) over 150 mg/l, age over 65 years, diabetes, gangrene of the gallbladder and an abscess as risk factors for conversion. Complications were experienced by 67 (18.0%) patients. Multivariate logistic regression identified age over 65 years, male gender, impaired renal function and conversion as risk factors for complications.

**Conclusions:**

Advanced cholecystitis with high CRP, gangrene or an abscess increase the risk of conversion. The risk of postoperative complications is higher after conversion. Early identification and treatment of acute calculous cholecystitis might reduce the number of patients with advanced cholecystitis and thus improve outcomes.

**Electronic supplementary material:**

The online version of this article (doi:10.1186/s13017-016-0111-4) contains supplementary material, which is available to authorized users.

## Background

Laparoscopic cholecystectomy (LC) is the standard treatment for acute cholecystitis [1, 2]. LC has been linked to a lower complication rate and shorter postoperative hospital stay compared with open cholecystectomy (OC) [3, 4]. Performing early cholecystectomy on patients admitted for acute cholecystitis is preferable to postponing the operation to be performed when the acute phase is over, since an early procedure has been recognized to shorten postoperative hospital stay and lower hospital care expenses [5, 6].

Gallbladder disease is among the leading causes for hospital admission for acute abdomen among adults and the most common indication for abdominal surgery in the elderly [7, 8]. In situations when LC is unsafe the surgeon might have to convert to an open procedure. The risk of conversion is higher in LC for acute cholecystitis than it is in an elective procedure [9]. The risk of conversion for patients undergoing LC for acute cholecystitis has been linked to male gender, age, previous endoscopic retrograde cholangiopancreatography (ERCP), a non-palpable gallbladder, elevated C-reactive protein (CRP) and white blood cell count (WBCC), gangrenous inflammation and the experience of the operating surgeon [10–13]. Conservative treatment with antibiotics and delaying the procedure to be performed after the acute phase has shown no change in conversion and complication rates [5, 14]. Patients who have had to undergo conversion have had more complications, which have led to further operations and a longer postoperative hospital stay [10].

Accounting for risk factors for conversion and complications is important when planning the procedure and deciding on whom to assign to perform the cholecystectomy. Experienced surgeons have been shown to have a lower complication rate for LC compared with surgeons in training [11]. Informing the patient about the procedure and the risk of complications is likewise important.

The aim of this study was to identify risk factors associated with conversion in patients with acute calculous cholecystitis. The risk factors for postoperative complications and the most common complications were also to be identified.

## Methods

### Patients and data collection

The study was a retrospective analysis of consecutive patients who had undergone emergent cholecystectomy in Meilahti Hospital, a university teaching hospital, from January 2013 till December 2014. A total of 499 patients were identified from the operating room database by procedure codes for LC and OC and International Classification of Diseases codes for acute cholecystitis. Fifty-four patients were excluded due to acalculous cholecystitis, and 33 due to missing signs of acute inflammation on the removed gallbladder described by the operating surgeon. Nine excluded patients received cholecystectomy during the treatment of another disease that required hospital care and three patients were excluded due to gallbladder malignancy. Twenty-seven patients received OC and were excluded. The remaining 373 patients with acute calculous cholecystitis were included into analysis. Acute calculous cholecystitis was defined as stones visible on preoperative imaging or during gallbladder removal and signs of acute cholecystitis described by the surgeon during the operation.

The preoperative diagnosis of acute cholecystitis had been reached by clinical assessment (tenderness in right upper quadrant, Murphy’s sign, fever), laboratory findings (elevated CRP and WBCC) and radiological signs of acute cholecystitis on imaging (thickened gallbladder wall, stones, enlarged gallbladder, edema, abscess).

More than 70 variables concerning personal data, clinical, laboratory, radiographic and intraoperative findings, the procedure and possible complications were collected directly or manually from the patient records. Complications were rated according to the Clavien-Dindo classification and the comprehensive complication index was calculated [15]. It was also specified what the complications were and how they were treated. Appropriate permissions to conduct the study were obtained from the hospital review board.

### Statistical analysis

The analysis of the data was conducted by SPSS Statistics v.22 for Mac OS X (IBM, Armonk, NY). Proportions were reported for categorical variables. Median, interquartile range and range was reported for continuous variables. *P* value was obtained using either Chi-square test or Fischer’s exact test for categorical variables and Mann-Whitney *U* test for continuous variables. Continuous variables used for multivariate analysis (CRP, WBCC, age, duration of surgery) were made categorical using cut off values determined from receiver operating characteristic (ROC) curves (Additional files 1: Figures S1, Additional files 2: Figures S2, Additional files 3: Figures S3 and Additional files 4: Figures S4). Optimal cut off values were defined as the values showing the highest sum of sensitivity and specificity on the ROC curves. Variables with a *P* value of <0.05 were considered statistically significant and were considered for inclusion in the multivariate analysis. A stepwise forward conditional approach of binary logistic regression was used to identify both risk factors for conversion and complications at a 0.05 significance level.

## Results

LC was initiated in 373 patients of which 84 (22.5%) were converted. Ultrasound (*n* = 301, 80.7%) was the main choice of imaging in patients with clinical suspicion of acute cholecystitis. Computed tomography (*n* = 127, 34.0% was mainly used in patients who presented with severe or diffuse symptoms, and magnetic resonance imaging (*n* = 93, 24.9%) was mainly used in patients with suspicion of bile duct stones in addition to cholecystitis. There were signs of acute calculous cholecystitis on imaging in 314 patients (84.2%) and of acute cholecystitis without radiologically visible stones in 46 patients (12.3%). Gallstones without signs of inflammation were visible in 12 patients (3.2%), and one patient did not undergo any preoperative imaging since the patient was in line for an elective cholecystectomy due to symptomatic gallstones and there was a strong suspicion of acute cholecystitis based on clinical and laboratory findings. On histopathological examination acute cholecystitis was found in 181 (48.5%), gangrenous cholecystitis in 98 (26.3%), acute on chronic cholecystitis in 48 (12.9%) and chronic cholecystitis in 46 (12.3%) patients.

### Conversion

Univariate analysis was performed in order to identify risk factors for conversion (Table 1). A multivariate analysis was conducted separately for preoperative risk factors only and for all risk factors for conversion, and the results are presented in Table 2. The most common reasons for conversion were severe inflammation reported in 47 patients (56.0%) and difficulty with identification of anatomy in 17 patients (20.2%).

**Table 1:** Univariate analysis of risk factors for conversion

	Conversion (*n* = 84 22.5 %)^a^	LC (*n* = 289)	Total (*n* = 373)	OR (95% CI)	*P*
Gender; Male	48 (25.4%)	141 (48.8%)	189 (50.7%)	1.4 (0.9–2.3)	0.178
Age in years, median (range)	70 (30–92)	61 (20–94)	63(20–94)	NA	<0.001
Heart failure	7 (29.2%)	17 (5.9%)	24 (6.4%)	1.5 (0.6–3.6)	0.420
Impaired renal function	2 (18.2%)	9 (3.1%)	11 (2.9%)	0.8 (0.2–3.6)	1.000
Diabetes	23 (33.8%)	45 (15.6%)	68 (18.2%)	2.0 (1.2–3.6)	0.014
Previous laparotomy on the upper abdomen	8 (40.0%)	12 (4.2%)	20 (5.4%)	2.4 (1.0–6.2)	0.054
Previous laparotomy on the lower abdomen^b^	21 (23.9%)	67 (24.3%)	88 (24.6%)	1.1 (0.6–1.9)	0.805
Previous laparoscopic surgery on the abdomen^b^	7 (29.2%)	17 (6.2%)	24 (6.7%)	1.4 (0.6–3.6)	0.450
No previous surgeries	52 (21.3%)	192 (66.4%)	244 (65.4%)	0.8 (0.5–1.4)	0.442
Duration of symptoms before admission^c^					
< 24 h	18 (14.4%)	106 (40.2%)	124 (36.9%)	0.5 (0.3–0.9)	0.018
24 h – 48 h	13 (27.1%)	35 (13.3%)	48 (14.3%)	1.4 (0.7–2.9)	0.302
48 h – 72 h	16 (25.0%)	48 (18.2%)	64 (19.0%)	1.3 (0.7–2.4)	0.439
> 72 h	25 (25.3%)	75 (28.4%)	100 (29.8%)	1.3 (0.8–2.3)	0.299
Hours from admission to surgery, median (range, interquartile range)	26 (3–109, 18–42)	29 (3–144, 20–48)	29 (3–144, 20–47)	NA	0.156
Total duration from symptoms onset to surgery^c^					
< 24 h	12 (21.4%)	44 (16.7%)	56 (16.7%)	1.0 (0.5–2.0)	1.000
24 h - 48 h	8 (16.3%)	41 (15.5%)	49 (14.6%)	0.7 (0.3–1.5)	0.346
48 h - 72 h	16 (21.3%)	59 (22.3%)	75 (22.3%)	1.0 (0.5–1.9)	0.982
> 72 h	35 (22.6%)	120 (45.5%)	155 (46.3%)	1.2 (0.7–2.0)	0.564
Clinical findings					
Pain in right upper quadrant	82 (23.1%)	273 (94.5%)	355 (95.2%)	2.4 (0.5–10.7)	0.235
Guarding	41 (25.6%)	119 (41.2%)	160 (42.9%)	1.4 (0.8–2.2)	0.213
Signs of generalized peritonitis	1 (50.0%)	1 (0.3%)	2 (0.5%)	3.5 (0.2–56.0)	0.400
Palpable gallbladder	6 (30.0%)	14 (4.8%)	20 (5.4%)	1.5 (0.6–4.1)	0.410
Preoperative laboratory data, median of highest results (range, interquartile range)^d^					
CRP (mg/l)	215 (3–471, 128–299)	123 (3–524, 58–214)	145 (3–524, 66–244)	NA	<0.001
WBCC (10^9^/l)	14 (7–38, 12–18)	13 (2.5–32, 10–16)	13 (2.5–38, 10–17)	NA	0.018
ALAT (U/l)	27 (5–222, 17–48)	30 (4–705, 18–63)	29 (4–705, 18–57)	NA	0.214
AFOS (U/l)	85 (48–371, 66–126)	80 (24–621, 61–112)	81 (24–621, 62–113)	NA	0.075
Bilirubin (μmol/l)	15 (4–121, 9–27)	15 (2–230, 9–24)	15 (2–230, 9–24)	NA	0.668
Radiographic findings^e^					
Abscess	1 (100.0%)	0	1 (0.3%)	NA	0.226
Free fluid	9 (36.0%)	16 (5.6%)	25 (6.7%)	2.0 (0.9–4.8)	0.097
Thickened gallbladder wall	72 (23.2%)	239 (83.0%)	311 (83.6%)	1.2 (0.6–2.4)	0.552
Preoperative ERCP	3 (17.6%)	14 (4.8%)	17 (4.6%)	0.7 (0.2–2.6)	0.773
Surgical findings					
Abscess	10 (66.7%)	5 (1.7%)	15 (4.0%)	7.7 (2.5–23.1)	<0.001
Perforated gallbladder	12 (33.3%)	24 (8.3%)	36 (9.7%)	1.8 (0.9–3.9)	0.102
Gallbladder perforation during surgery	43 (23.0%)	144 (49.8%)	187 (50.1%)	1.1 (0.7–1.7)	0.826
Gangrene of the gallbladder identified by surgeon	58 (43.0%)	77 (26.6%)	135 (36.2%)	6.4 (3.6–10.4)	<0.001
Removal of stones from common bile duct	0	0	0	NA	NA
Lead surgeon specialist	32 (25.2%)	94 (32.5%)	126 (33.8%)	1.3 (0.8–2.1)	0.342
Assistant present	53 (29.9%)^f^	124 (42.9%)	177 (48.1%)	2.7 (1.6–4.6)	<0.001

*NA* Not applicable, *LC* Laparoscopic cholecystectomy, *OR* Odds ratio, *CRP* C-reactive protein, *WBCC* White blood cell count, *ERCP* Endoscopic retrograde cholangiopancretography, *ALAT* Alanine transferase, *AFOS* Alkaline phosphatase

^a^percentages show the proportion of patients with specified risk factor

^b^Fifteen patients had mentions of an appendectomy in their patient journals, but it was not specified whether the procedure was laparoscopic or open

^c^Duration of symptoms missing from 25 patients in LC and 12 in conversion

^d^CRP missing from one patient, ALAT missing from seven patients, AFOS missing from eight patients, bilirubin missing from 18 patients

^e^A total of 239 patients had an ultrasound, 65 had computed tomography (CT) and 62 had both. Magnetic resonance cholangiopancreatography (MRCP) was used in 93 patients. One patient did not undergo any imaging

^f^Assistant present prior to conversion, info on timing of assistant arrival missing in five cases

**Table 2:** Independent risk factors for conversion based on stepwise forward logistic regression

Risk factor	OR (95% CI)	*P*
Analysis of preoperative risk factors only		
CRP over 150 mg/ml	3.0 (1.8–5.0)	<0.001
Diabetes	1.8 (1.0–3.3)	0.045
Analysis including both preoperative and intraoperative risk factors		
Abscess	9.2 (2.7–31.1)	<0.001
Age over 65 years	1.9 (1.1–3.3)	0.023
Gangrene of the gallbladder	5.9 (3.4–10.2)	<0.001

The following preoperative findings were included in the stepwise forward logistic regression analysis of risk factors for conversion: age over 65 years, previous laparotomy on the upper abdomen, diabetes, CRP over 150 mg/ml and WBCC over 13x10^9^/l. Gangrene of the gallbladder and abscess were added for the stepwise analysis of all risk factors. CRP C-reactive protein, WBCC white blood cell count

### Complications

A univariate analysis of risk factors for postoperative complications is presented in Table 3. Risk factors for complications identified by multivariate analysis are presented in Table 4. Sixty-seven (18.0%) patients experienced an overall of 83 complications. The complication rates were 14.5 and 29.8% for LC and conversion respectively (*p* < 0.001). The 67 patients are grouped in Table 5 according to their most serious complication. The median comprehensive complication index for all patients with complications was 22.6 with an interquartile range of 20.9–26.2 and a range of 8.7–100. Twenty (5.4%) patients experienced complications that required surgical, endoscopic or radiological intervention. Three patients (0.8%) experienced a life threatening complication (grade IV) and five (1.3%) deaths (grade V) occurred.

**Table 3:** Univariate analysis of risk factors for complications

Risk factor	One or more postoperative complications *n* = 67 (18.0%)^a^	Patients without complications *n* = 306	OR (95% CI)	*P*
Gender: Male	44 (23.3%)	145	2.1 (1.2–3.7)	0.007
Age in years, median (range)	70 (30–92)	61 (20–94)	NA	0.001
Heart failure	7 (29.2%)	17	2.0 (0.8–5.0)	0.139
Impaired renal function	6 (54.5%)	5	5.9 (1.8–20.0)	0.006
Diabetes	18 (26.5%)	50	1.9 (1.0–3.5)	0.043
Previous laparotomy on the upper abdomen	8 (40.0%)	12	3.3 (1.3–8.5)	0.008
Previous laparotomy on the lower abdomen^b^	16 (18.2%)	72	1.0 (0.5–1.8)	0.944
Previous laparoscopic surgery on the abdomen^b^	2 (8.3%)	22	0.4 (0.1–1.7)	0.276
No previous surgeries	43 (17.7%)	200	1.0 (0.5–1.6)	0.854
Duration of symptoms before admission^c^				
< 24 h	18 (14.5%)	106	0.8 (0.4–1.4)	0.360
24 h – 48 h	11 (22.9%)	37	1.6 (0.7–3.3)	0.235
48 h – 72 h	10 (15.6%)	54	0.9 (0.4–1.9)	0.751
> 72 h	18 (18.0%)	82	1.1 (0.6–2.1)	0.742
Hours from admission to surgery, median (range, interquartile range)	26 (3–92, 16–43)	29 (4–144, 20–48)	NA	0.141
Duration from symptoms onset to surgery^c^				
< 24 h	9 (16.1%)	47	0.9 (0.4–2.0)	0.845
24 h - 48 h	9 (18.4%)	40	1.1 (0.5–2.5)	0.777
48 h - 72 h	14 (18.7%)	61	1.2 (0.6–2.3)	0.656
> 72 h	24 (15.5%)	131	0.8 (0.5–1.5)	0.575
Clinical findings				
Pain in right upper quadrant	65 (18.3%)	290	1.8 (0.4–8.0)	0.438
Guarding	33 (20.6%)	127	1.4 (0.8–2.3)	0.246
Signs of generalized peritonitis	0	2	NA	1.000
Palpable gallbladder	4 (20.0%)	16	1.2 (0.4–3.6)	0.767
Preoperative laboratory data, median of the highest results (range, interquartile range)^d^				
CRP (mg/l)	209 (4–477, 114–303)	131 (3–524, 62–232)	NA	<0.001
WBCC (10^9^/l)	14.4 (5.4–37.4, 10.8–18.5)	12.7 (2.5–38, 10–16.5)	NA	0.069
ALAT (U/l)	31 (5–473, 18–74)	28 (4–705, 18–51)	NA	0.439
AFOS (U/l)	91 (24–314, 68–118)	79 (26–621, 61–113)	NA	0.105
Bilirubin (μmol/l)	19 (4–92, 11–25)	14 (2–230, 9–24)	NA	0.139
Radiographic findings^e^				
Abscess	0	1	NA	1.000
Free fluid	7 (28.0%)	18	1.9 (0.7–4.7)	0.182
Thickened gallbladder wall	61 (19.6%)	250	2.2 (0.9–5.4)	0.069
Surgical findings and procedures				
Preoperative ERCP	3 (17.6%)	14	1.0 (0.3–3.5)	1.000
Abscess	5 (33.3%)	10	2.4 (0.8–7.2)	0.160
Perforated gallbladder	9 (25.0%)	27	1.6 (0.7–3.6)	0.234
Gallbladder perforation during surgery	38 (20.3%)	149	1.4 (0.8–2.4)	0.452
Gangrene of the gallbladder identified by surgeon	31 (23.0%)	104	1.7 (1.0–2.9)	0.058
Removal of stones from the common bile duct	0	0	NA	NA
Duration of surgery in minutes, median (range, interquartile range)	110 (60–196, 83–138)	98 (34–240, 74–123)	NA	0.023
Lead surgeon specialist	24 (19.0%)	102	1.1 (0.6–1.9)	0.697
Assistant present^f^	34 (19.2%)	143	1.2 (0.7–2.0)	0.540
Conversion	25 (29.8%)	59	2.5 (1.4–4.4)	0.001

*NA* Not applicable, *LC* Laparoscopic cholecystectomy, *OR* Odds ratio, *CRP* C-reactive protein, *WBCC* White blood cell count, *ERCP* Endoscopic retrograde cholangiopancretography, *ALAT* Alanine transferase, *AFOS* Alkaline phosphatase

^a^percentages show the proportion of patients with specified risk factor

^b^Fifteen patients had mentions of an appendectomy in their patient journals, but it was not specified whether the procedure was laparoscopic or open

^c^Duration of symptoms missing from 25 patients in LC and 12 in conversion

^d^CRP missing from one patient, ALAT missing from seven patients, AFOS missing from eight patients, bilirubin missing from 18 patients

^e^A total of 239 patients had an ultrasound, 65 had computed tomography (CT) and 62 had both. Magnetic resonance cholangiopancreatography (MRCP) was used in 93 patients. One patient did not undergo any imaging

^f^Assistant present prior to conversion, info on timing of assistant arrival missing in five cases

**Table 4:** Independent risk factors for complications based on stepwise forward logistic regression

Risk factor	OR (95% CI)	*P*
Age over 65 years	2.1 (1.2–3.6)	0.012
Male gender	2.1 (1.2–3.7)	0.013
Impaired renal function	4.8 (1.4–17.0)	0.015
Conversion	2.3 (1.3–4.1)	0.006

Variables included in the stepwise forward logistic regression analysis of risk factors for complications were age over 65 years, male gender, C-reactive protein over 150 mg/ml, diabetes, impaired renal function, previous laparotomy on the upper abdomen, duration of surgery over 90 min and conversion

**Table 5:** Classification of complications that occurred within 30 days from surgery according to the Clavien-Dindo classification and their treatment

Clavien-Dindo classification	LC (*n* = 289)	Conversion (*n* = 84)	Total (*n* = 373)	Treatment
Grade I-II	24 (8.3%)	15 (17.9%)	39 (10.5%)	
Infections				Antimicrobial medication
Pneumonia	8	4	12	
Superficial SSI	4	2	6^a^	
Urinary tract infection	1		1	
Other infection	1	2	3	
Arrhythmias	4		4	Antiarrhythmic medication
High blood pressure		2	2	Blood pressure medication
Perioperative MI	2		2	Medication
Respiratory insufficiency	1	1	2	Medication
Congestion	1	1	2	Medication
Urinary retention		1	1	Catheterisation
Postoperative delirium	1		1	Medication
Nausea		1	1	Antiemetic medication
Perihepatic hematoma		1	1	Follow-up
Wound hematoma	1		1	Change of dressings
Grade III a-b	13 (4.5%)	7 (8.3%)	20 (5.4%)	
Common bile duct stone	5		5	ERCP
Surgical site effusion	4		4	Drainage
Bile leak		3	3^a^	ERCP, stenting
Deep SSI; abscess	2	1	3	Drainage
Wound dehiscence		2	2	Re-suturation
Bleeding from urinary catheter	1		1	Bladder washout
Intra-abdominal bleeding	1		1	Laparotomy
Pneumonia; pleural effusion		1	1	Thoracocentesis
Grade IV a-b	2 (0.7%)	1 (1.2%)	3 (0.8%)	
Respiratory failure	2	1	3	Intubation, CPAP
Grade V	3 (1.0%)	2 (2.4%)	5 (1.3%)	
Sepsis	1		1	
Pneumonia		1	1	
Heart failure	1		1	
Anoxic brain injury during induction of anaesthesia		1	1	
Renal failure	1		1	
Total	42 (14.5%)	25 (29.8%)	67 (18.0%)	

The most serious complication was classified in patients who experienced multiple complications *LC* Laparoscopic cholecystectomy, *SSI* Surgical site infection, *ERCP* Endoscopic retrograde cholangiopancreatography, *CPAP* Continuous positive airway pressure, *MI* Myocardial infarction

^a^The numbers of superficial SSIs and bile leaks are reported lower here than in the text (seven respectively four), since only the most serious complication in patients with multiple complications was categorised in this table. The most serious complication in the fourth patient with a bile leak was a wound dehiscence (Clavien-Dindo grade IIIb)

Of the total number of 83 complications, the most common complications were pneumonia, which occurred in 14 patients (3.8%), a superficial surgical site infection (SSI) in seven patients (1.9%) and a retained common bile duct stone in five patients (1.3%). A bile leak occurred in four patients (1.1%). Two of the bile leaks were from the cystic duct, one from the main bile duct and one was undefined. None of the bile leaks occurred in patients who had undergone LC. The patients who died had a mean age of 81 years (range 70–92) and they all had at least two comorbidities of which one was a cardiovascular comorbidity. The American Society of Anesthesiologists (ASA) classification was IV for four patients and III for one patient. Of the three deaths in the LC group one was from the worsening of heart failure, another from the worsening of renal failure and the third from sepsis. The two deaths among converted patients were caused by postoperative pneumonia and failure of intubation during the induction of anaesthesia, leading to an anoxic brain injury.

## Discussion

LC has become the standard procedure for managing acute calculous cholecystitis. The main concerns are with safety and feasibility as reflected in the risk of conversion to open cholecystectomy as well as the risk of postoperative complications, especially bile duct injuries. Our study focused on the risk factors for conversion and postoperative complications.

### Conversion

Age over 65 years, diabetes and CRP over 150 mg/l were identified as independent preoperative risk factors for conversion. Complications of severe inflammation like gangrene of the gallbladder and an abscess identified by the surgeon were also recognized as risk factors in the multivariate analysis including both preoperative and intraoperative findings (Table 2). Diabetes and CRP over 150 mg/l were however not of significant value in this analysis, which might speak for a correlation between diabetes and the development of gangrene and an abscess leading to high CRP levels. Studies have indeed found that diabetes increases the risk of development of gangrenous cholecystitis and that gangrene increases the risk of conversion [13, 16, 17]. Gangrene and an abscess might however be hard to recognize prior to the operation and therefore CRP levels and history of diabetes might be of better use when estimating the difficulty of the planned procedure.

High age, diabetes and CRP have been recognized as risk factors for conversion by other studies as well [10, 12, 13, 18]. Age as a risk factor has been speculated to be related to a longer history of gallbladder disease, masked symptoms and patient delay [12, 18]. History of previous abdominal surgeries and male gender have also been linked to conversion [18]. Seven (8.3%) patients were converted due to adhesions from previous surgeries, but history of previous abdominal surgeries was not recognized as a risk factor in this study and neither was male gender. The presence of an assistant was associated with conversion in the univariate analysis, which is probably a result from assistants being called to particularly challenging surgeries. Hence we did not consider the presence of an assistant as a risk factor for conversion.

Early cholecystectomy is recommended over conservative treatment followed by delayed cholecystectomy [14, 19]. The optimal time point for cholecystectomy resulting in the lowest conversion and complication rates is still under debate [2]. Some have found that surgery within 48 h from admission lowers the complication rate, whilst others have concluded that cholecystectomy within 5 days of admission yields as good as results in conversion and complications as surgery performed as soon as scheduling allows [20, 21]. Also, a recent randomized trial found that early LC results in lower morbidity and hospital stay compared to delayed cholecystectomy even in acute cholecystitis with symptoms over 72 h prior to admission [22].

No correlation between the time from admission to surgery or the total duration of symptoms and conversion or complications was however documented in this study. This might have been caused by a selection bias resulting from patients with a clinically more severe condition being operated on earlier. Also the initial onset of symptoms might have been hard to notice by elderly patients with several comorbidities. After diagnosis of cholecystitis antibiotics were initiated, which might have slowed down the progression of cholecystitis. Prehospital delay of less than 24 h from symptoms onset was associated with the lowest conversion rate, which might tell us that cholecystitis without any treatment is of higher significance than the in-hospital delay. It is also possible that the inflammation progresses individually and that time does not seemingly have a great impact on the development of the inflammatory process.

### Complications

Age over 65 years, male gender, impaired renal function and surgery finished as open cholecystectomy were identified as independent risk factors for complications. The overall complication rate of 18.0% falls within complication rates of 9 – 20% reported by other studies [12, 13]. The complication rate after conversion was significantly higher than after LC. Since higher age, diabetes and advanced infection were associated with conversion it is possible that these factors also contribute to increased postoperative complications. Furthermore, wound complications like wound infections and ruptures were more common after conversion to open surgery. Age, male gender and gangrene have been recognized as risk factors for complications by other studies on acute cholecystitis [12, 13, 17]. Studies with patients operated on both electively and emergently have also recognized age, male gender and conversion as risk factors for complications [23, 24].

The overall bile duct injury (BDI) rate in this study was 1.1%, with a rate of 4.8% for cholecystectomies finished as open surgeries. However, most bile duct injuries were Strasberg classification type A, and all injuries were managed endoscopically [25]. None of the BDIs in this study occurred in patients who received LC, which reflects on the safety of LC, but might also be a result from difficult procedures being converted before BDIs could occur. This is supported by the rather high conversion rate in our study. Other studies have reported BDI rates of 0.62–0.9% for LC and 0.38–1.24% for OC [26–28]. These studies were however not homogenous for acute cholecystitis for which the risk of BDI has been reported as twice as high compared with patients who undergo cholecystectomy electively [29].

It was predicted that the BDI rates after LC would become lower as the procedure became more common, but according to some studies this prediction has not yet been fulfilled [30]. There are also results on the opposite trend – BDI injuries having become more common after OC – which raises concern on surgeons in training not learning appropriate technique for OC in the laparoscopic era [27]. Patients chosen for conversion or direct OC are however often suffering from a more severe inflammation that makes the tissues more prone to rupture and hence patients who have their surgery finished as an open procedure might be at risk for BDI due to inflamed, rupture-prone tissues rather than inadequate surgical technique. Some studies comparing the results on surgeons in training versus specialists performing cholecystectomy have concluded that the overall complication rate is higher for surgeons in training [11]. Our study did not show such a correlation, but this might be due to a bias resulting from clinically more severe cases being assigned to specialists. Prospective randomized trials are naturally unethical to perform since patients should always be offered the best care available.

Mortality (1.3%) in the present study was somewhat higher than mortality rates of 0.7–1.1% reported by other studies [12, 31]. The patients who suffered a mortal complication in this study had several comorbidities and were clinically considered high risk surgical patients. Treatment options for severe acute cholecystitis apart from cholecystectomy consist of antibiotics and interval cholecystectomy or the use of percutaneous transhepatic cholecystostomy (PTHC) possibly followed by cholecystectomy. Currently there are no results on the impact of interval cholecystectomy or PTHC for subgroups of high risk surgical patients. To determine the best treatment in such patients a randomized controlled trial has been initiated in the Netherlands where the use of LC and PTHC in high risk patients are to be compared [32].

### Limitations

Like all retrospective review studies this study has its limits. Data concerning body mass index, which might have been associated with conversion, was missing from many patients and was not included in the study. Data on symptoms duration was also missing from many patients and hence it was not included in the multivariate analysis. The study is also limited by its sample size. Different risk factors might correlate with different complications and the severity of complications, but such a correlation cannot be evaluated with a limited sample size.

## Conclusions

Early LC is safe and feasible in the treatment of acute calculous cholecystitis. The risk of postoperative complications is increased by risk factors like male gender, high age and impaired renal function and conversion to open surgery. Of these factors the only one that can be influenced is conversion. Manifestations of advanced cholecystitis like high CRP, gangrene of the gallbladder or abscess formation increase the risk of conversion to open cholecystectomy. Early identification and treatment of acute calculous cholecystitis might lower the number of patients with advanced cholecystitis and thus reduce the amount of converted patients and postoperative complications. When LC cannot be performed safely conversion should be initiated to minimize the risk of bile duct injuries. Also enough attention should be paid to surgeons in training learning appropriate technique for performing open cholecystectomy.

## Additional files

Additional file 1: Figure S1.
**a**) Receiver operating characteristic (ROC) curve for C-reactive protein (CRP) in converted patients. CRP level of 150 mg/ml yields sensitivity of 0.69 and specificity of 0.58. Area under the curve (AUC) 0.67. **b**) ROC curve for CRP in patients with complications. CRP level of 150 mg/ml yields sensitivity of 0.64 and specificity of 0.55. AUC 0.64. (PDF 113 kb)
Additional file 2: Figure S2.
**a**) Receiver operating characteristic (ROC) curve for age in converted patients. Age of 65 years yields sensitivity of 0.58 and specifity of 0.57. Area under the curve (AUC) 0.63. **b**) ROC curve for age in patients with complications. Age of 65 years yields sensitivity of 0.63 and specificity of 0.59. AUC 0.63. (PDF 131 kb)
Additional file 3: Figure S3. Receiver operating characteristic (ROC) curve for white blood cell count (WBCC) in converted patients. WBCC of 13x10^9/l yields a sensitivity of 0.62 and specificity of 0.54. Area under the curve (AUC) 0.59. (PDF 106 kb)
Additional file 4: Figure S4. Receiver operating characteristic (ROC) curve for duration of surgery in patients with complications. Surgery duration of 90 min yields a sensitivity of 0.72 and specificity of 0.40. Area under the curve (AUC) 0.59. (PDF 88 kb)

## Abbreviations

BDI: Bile duct injury
CRP: C-reactive protein
ERCP: Endoscopic retrograde cholangiopancreatography
LC: Laparoscopic cholecystectomy
OC: Open cholecystectomy
PTHC: Percutaneous transhepatic cholecystostomy
ROC: Receiver operating characteristic
SSI: Surgical site infection
WBCC: White blood cell count

## References

[1] Coccolini F, Catena F, Pisano M, Gheza F, Fagiuoli S, Di Saverio S (2015). Open versus laparoscopic cholecystectomy in acute cholecystitis. Systematic review and meta-analysis. Int J Surg.

[2] Ansaloni L, Pisano M, Coccolini F, Peitzmann AB, Fingerhut A, Catena F (2016). 2016 WSES guidelines on acute calculous cholecystitis. World J Emerg Surg.

[3] Kiviluoto T, Sirén J, Luukkonen P, Kivilaakso E (1998). Randomised trial of laparoscopic versus open cholecystectomy for acute and gangrenous cholecystitis. Lancet.

[4] Boo YJ, Kim WB, Kim J, Song TJ, Choi SY, Kim YC (2007). Systemic immune response after open versus laparoscopic cholecystectomy in acute cholecystitis: A prospective randomized study. Scand J Clin Lab Invest.

[5] Gurusamy K, Samraj K, Gluud C, Wilson E, Davidson BR (2010). Meta-analysis of randomized controlled trials on the safety and effectiveness of early versus delayed laparoscopic cholecystectomy for acute cholecystitis. Br J Surg.

[6] Wilson E, Gurusamy K, Gluud C, Davidson BR (2010). Cost-utility and value-of-information analysis of early versus delayed laparoscopic cholecystectomy for acute cholecystitis. Br J Surg.

[7] Miettinen P, Pasanen P, Lahtinen J, Alhava E (1996). Acute abdominal pain in adults. Ann Chir Gynaecol.

[8] Ukkonen M, Kivivuori A, Rantanen T, Paajanen H (2015). Emergency Abdominal Operations in the Elderly: A Multivariate Regression Analysis of 430 Consecutive Patients with Acute Abdomen. World J Surg.

[9] Giger UF, Michel J-M, Opitz I, Th Inderbitzin D, Kocher T, Krahenbuhl L (2006). Risk factors for perioperative complications in patients undergoing laparoscopic cholecystectomy: analysis of 22,953 consecutive cases from the Swiss Association of Laparoscopic and Thoracoscopic Surgery database. J Am Coll Surg.

[10] Dominguez LC, Rivera A, Bermudez C, Herrera W (2011). Analysis of factors for conversion of laparoscopic to open cholecystectomy: a prospective study of 703 patients with acute cholecystitis. Cir Esp.

[11] Hobbs MS, Mai Q, Knuiman MW, Fletcher DR, Ridout SC (2006). Surgeon experience and trends in intraoperative complications in laparoscopic cholecystectomy. Br J Surg.

[12] Wevers KP, van Westreenen HL, Patijn GA (2013). Laparoscopic cholecystectomy in acute cholecystitis: C-reactive protein level combined with age predicts conversion. Surg Laparosc Endosc Percutan Tech.

[13] Eldar S, Sabo E, Nash E, Abrahamson J, Matter I (1997). Laparoscopic Cholecystectomy for Acute Cholecystitis: Prospective Trial. World J Surg.

[14] Gutt CN, Encke J, Koninger J, Harnoss J-C, Weigand K, Kipfmuller K (2013). Acute cholecystitis: early versus delayed cholecystectomy, a multicenter randomized trial (ACDC study, NCT00447304). Ann Surg.

[15] Dindo D, Demartines N, Clavien P-A (2004). Classification of surgical complications: a new proposal with evaluation in a cohort of 6336 patients and results of a survey. Ann Surg.

[16] Bourikian S, Anand RJ, Aboutanos M, Wolfe LG, Ferrada P (2015). Risk factors for acute gangrenous cholecystitis in emergency general surgery patients. Am J Surg.

[17] Ganapathi AM, Speicher PJ, Englum BR, Perez A, Tyler DS, Zani S (2015). Gangrenous cholecystitis: a contemporary review. J Surg Res.

[18] Simopoulos C, Botaitis S, Polychronidis A, Tripsianis G, Karayiannakis AJ (2005). Risk factors for conversion of laparoscopic cholecystectomy to open cholecystectomy. Surg Endosc.

[19] Lo CM, Liu CL, Fan ST, Lai EC, Wong J (1998). Prospective randomized study of early versus delayed laparoscopic cholecystectomy for acute cholecystitis. Ann Surg.

[20] Banz V, Gsponer T, Candinas D, Guller U (2011). Population-based analysis of 4113 patients with acute cholecystitis: defining the optimal time-point for laparoscopic cholecystectomy. Ann Surg.

[21] Chandler CF, Lane JS, Ferguson P, Thompson JE, Ashley SW (2000). Prospective evaluation of early versus delayed laparoscopic cholecystectomy for treatment of acute cholecystitis. Am Surg.

[22] Roulin D, Saadi A, Di Mare L, Demartines N, Halkic N (2016). Early Versus Delayed Cholecystectomy for Acute Cholecystitis, Are the 72 hours Still the Rule?. A Randomized Trial Ann Surg.

[23] Murphy MM, Ng S-C, Simons JP, Csikesz NG, Shah SA, Tseng JF (2010). Predictors of Major Complications after Laparoscopic Cholecystectomy: Surgeon, Hospital, or Patient?. J Am Coll Surg.

[24] Roslyn JJ, Binns GS, Hughes EF, Saunders-Kirkwood K, Zinner MJ, Cates JA (1993). Open cholecystectomy. A contemporary analysis of 42,474 patients. Ann Surg.

[25] Strasberg SM, Hertl M, Soper NJ (1995). An analysis of the problem of biliary injury during laparoscopic cholecystectomy. J Am Coll Surg.

[26] Diamantis T, Tsigris C, Kiriakopoulos A, Papalambros E, Bramis J, Michail P (2005). Bile duct injuries associated with laparoscopic and open cholecystectomy: an 11-year experience in one institute. Surg Today.

[27] Karvonen J, Salminen P, Grönroos JM (2011). Bile duct injuries during open and laparoscopic cholecystectomy in the laparoscopic era: alarming trends. Surg Endosc.

[28] Viste A, Horn A, Ovrebo K, Christensen B, Angelsen J-H, Hoem D (2015). Bile duct injuries following laparoscopic cholecystectomy. Scand J Surg.

[29] Tornqvist B, Waage A, Zheng Z, Ye W, Nilsson M (2016). Severity of Acute Cholecystitis and Risk of Iatrogenic Bile Duct Injury During Cholecystectomy, a Population-Based Case-Control Study. World J Surg.

[30] Slater K, Strong RW, Wall DR, Lynch SV (2002). Iatrogenic bile duct injury: the scourge of laparoscopic cholecystectomy. ANZ J Surg.

[31] Scollay JM, Mullen R, McPhillips G, Thompson AM (2011). Mortality associated with the treatment of gallstone disease: a 10-year contemporary national experience. World J Surg.

[32] Kortram K, van Ramshorst B, Bollen TL, Besselink MGH, Gouma DJ, Karsten T (2012). Acute cholecystitis in high risk surgical patients: percutaneous cholecystostomy versus laparoscopic cholecystectomy (CHOCOLATE trial): study protocol for a randomized controlled trial. Trials.

