# Supplementary material for: Laparoscopic cholecystectomy for acute calculous cholecystitis: a retrospective study assessing risk factors for conversion and complications
Source: World J Emerg Surg. 2016 Nov 16;11:54. doi: 10.1186/s13017-016-0111-4 (PMC5112701; doi:10.1186/s13017-016-0111-4)
Supplement: Additional file 1: Figure S1. — a) Receiver operating characteristic (ROC) curve for C-reactive protein (CRP) in converted patients. CRP level of 150 mg/ml yields sensitivity of 0.69 and specificity of 0.58. Area under the curve (AUC) 0.67. b) ROC curve for CRP in patients with complications. CRP level of 150 mg/ml yields sensitivity of 0.64 and specificity of 0.55. AUC 0.64. (PDF 113 kb) [file 13017_2016_111_MOESM1_ESM.pdf]

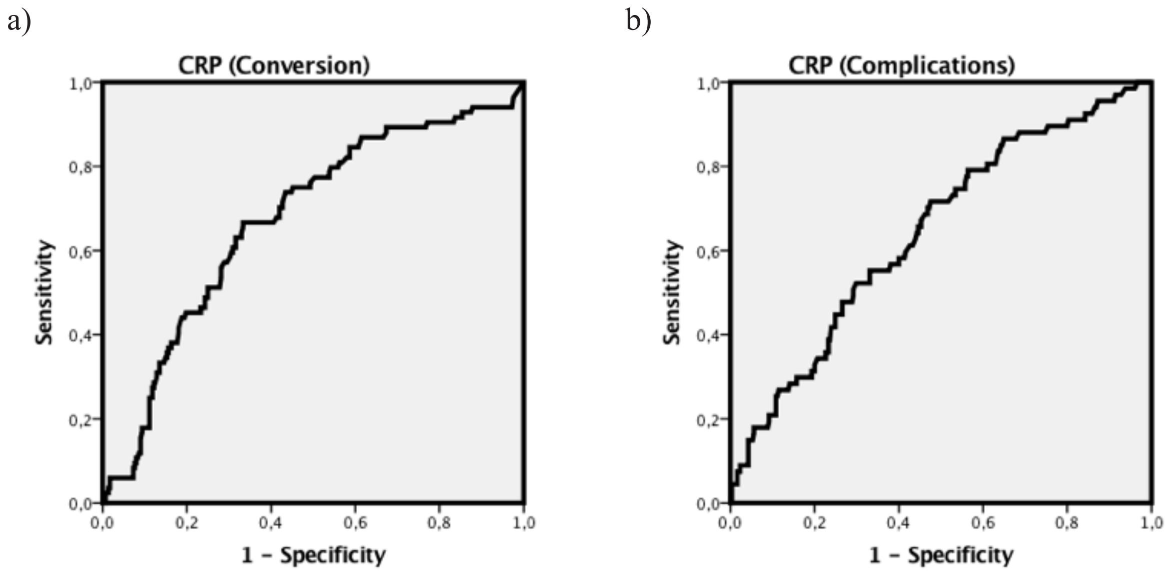

Figure 1
a) Receiver operating characteristic (ROC) curve for C-reactive protein (CRP) in converted patients. CRP level of 150 mg/ml yields sensitivity of 0.69 and specificity of 0.58. Area under the curve (AUC) 0.67.
b) ROC curve for CRP in patients with complications. CRP level of 150 mg/ml yields sensitivity of 0.64 and specificity of 0.55. AUC 0.64.
