# Supplementary material for: Laparoscopic cholecystectomy for acute calculous cholecystitis: a retrospective study assessing risk factors for conversion and complications
Source: World J Emerg Surg. 2016 Nov 16;11:54. doi: 10.1186/s13017-016-0111-4 (PMC5112701; doi:10.1186/s13017-016-0111-4)
Supplement: Additional file 2: Figure S2. — a) Receiver operating characteristic (ROC) curve for age in converted patients. Age of 65 years yields sensitivity of 0.58 and specifity of 0.57. Area under the curve (AUC) 0.63. b) ROC curve for age in patients with complications. Age of 65 years yields sensitivity of 0.63 and specificity of 0.59. AUC 0.63. (PDF 131 kb) [file 13017_2016_111_MOESM2_ESM.pdf]

a) b)

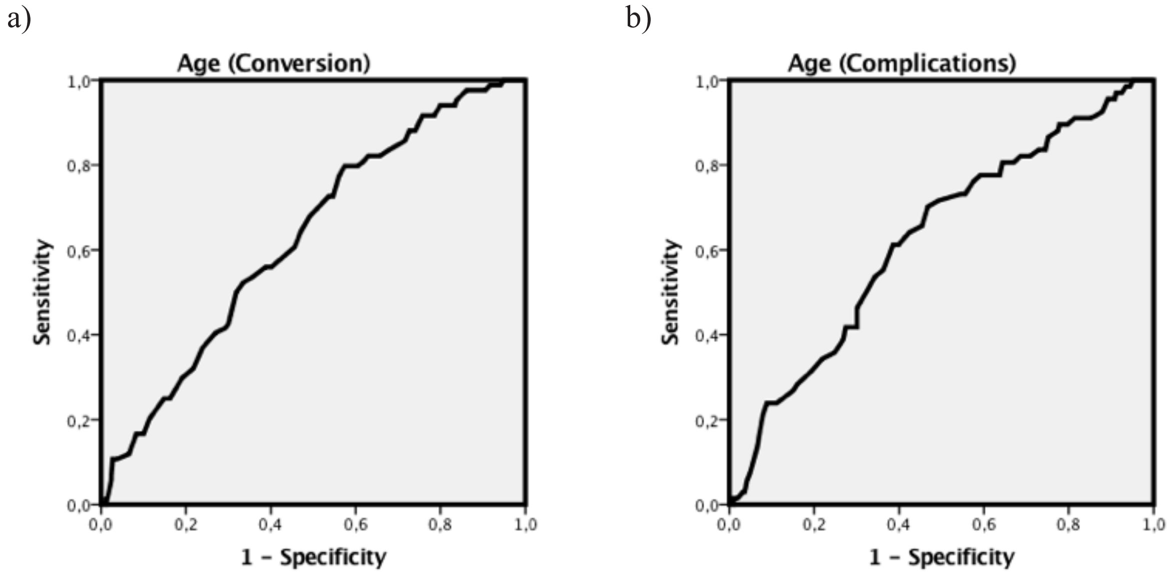


**Figure 2**
**a)** Receiver operating characteristic (ROC) curve for age in converted patients. Age of 65 years yields sensitivity of 0.58 and specifity of 0.57. Area under the curve (AUC) 0.63.
**b)** ROC curve for age in patients with complications. Age of 65 years yields sensitivity of 0.63 and specificity of 0.59. AUC 0.63.
