# Supplementary material for: Laparoscopic cholecystectomy for acute calculous cholecystitis: a retrospective study assessing risk factors for conversion and complications
Source: World J Emerg Surg. 2016 Nov 16;11:54. doi: 10.1186/s13017-016-0111-4 (PMC5112701; doi:10.1186/s13017-016-0111-4)
Supplement: Additional file 3: Figure S3. — Receiver operating characteristic (ROC) curve for white blood cell count (WBCC) in converted patients. WBCC of 13x10^9/l yields a sensitivity of 0.62 and specificity of 0.54. Area under the curve (AUC) 0.59. (PDF 106 kb) [file 13017_2016_111_MOESM3_ESM.pdf]

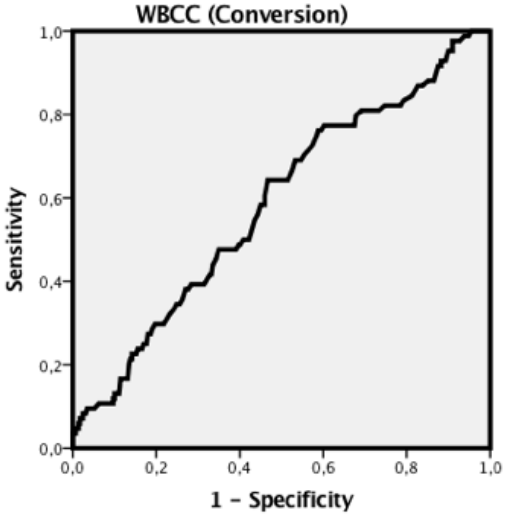

Figure 3
Receiver operating characteristic (ROC) curve for white blood cell count (WBCC) in converted patients. WBCC of 13x10^9/l yields a sensitivity of 0.62 and specificity of 0.54. Area under the curve (AUC) 0.59.
